# Family history of breast cancer and its association with disease severity and mortality

**DOI:** 10.1002/cam4.648

**Published:** 2016-01-22

**Authors:** Jennifer C. Melvin, Wahyu Wulaningsih, Zac Hana, Arnie D. Purushotham, Sarah E. Pinder, Ian Fentiman, Cheryl Gillett, Anca Mera, Lars Holmberg, Mieke Van Hemelrijck

**Affiliations:** ^1^ Faculty of Life Sciences and MedicineDivision of Cancer StudiesCancer Epidemiology GroupKing's College LondonLondonUnited Kingdom; ^2^ Faculty of Life Sciences and MedicineDivision of Cancer StudiesSection of Research OncologyKing's College LondonLondonUnited Kingdom; ^3^ Guy's and St. Thomas’ NHS Foundation TrustLondonUnited Kingdom; ^4^ Regional Cancer CentreUppsala/OrebroUppsalaSweden; ^5^ Research OncologyGuy's HospitalLondonUnited Kingdom; ^6^ Department of Surgical SciencesUppsala UniversityUppsalaSweden

**Keywords:** breast cancer, Family history, mortality, severity

## Abstract

A family history (FH) of breast cancer (BC) is known to increase an individual's risk of disease onset. However, its role in disease severity and mortality is less clear. We aimed to ascertain associations between FH of BC, severity and BC‐specific mortality in a hospital‐based cohort of 5354 women with prospective information on FH. We included women diagnosed at Guy's and St Thomas’ NHS Foundation Trust between 1975 and 2012 (*n* = 5354). BC severity was defined and categorized as good, moderate, and poor prognosis. Data on BC‐specific mortality was obtained from the National Cancer Registry and medical records. Associations between FH and disease severity or BC‐specific mortality were evaluated using proportional odds models and Cox proportional hazard regression models, respectively. Available data allowed adjustment for potential confounders (e.g., treatment, socioeconomic status, and ethnicity). FH of any degree was not associated with disease severity at time of diagnosis (adjusted proportional OR: 1.00 [95% CI: 0.85 to 1.17]), which remained true also after stratification by period of diagnosis. FH of BC was not associated with BC‐mortality HR: 0.99 (95% CI: 0.93 to 1.05). We did not find evidence to support an association between FH of BC and severity and BC‐specific mortality. Our results indicate that clinical management should not differ between women with and without FH, when the underlying mutation is unknown.

## Introduction

A family history of breast cancer (BC) is a known risk factor for the onset of disease 1, with an increased risk depending on the degree of family history 2, 3. However, the link between family history and severity at time of diagnosis is less well described. Further clarification of this association could help the implementation of appropriate guidelines for clinicians and patients, with regards to screening, diagnosis and treatment. The association between family history and BC severity is unclear, and existing literature is scarce. A study of 2256 BC patients with invasive operable BC concluded that patients with a family history had smaller tumors that were more likely to be estrogen receptor (ER) positive 4. However, a Swedish population‐based registry study found a nonsignificant improved prognosis in women with a family history of BC 5. Additionally, the association between family history and BC‐specific mortality is not well defined. A review by the Collaborative Group on Hormonal Factors in Breast Cancer, constituting 52 studies, did not find an association between family history and BC‐mortality 6 along with several others 7, 8, 9, 10, 11, 12, whereas other studies have found a positive or even an inverse association 5, 13, 14, 15, 16, 17, 18, 19, 20. One study of over 1200 young women (defined as those diagnosed before the age of 45 years) with invasive BC found that women with no family history had a reduced BC‐mortality. Interestingly, women with a first degree relation showed a 40% reduction in mortality risk, whereas women with only a second degree relation experienced similar results to women with no family history 17. On the contrary, another study reported no statistically significant difference between patients with a family history of BC with regard to the risk of mortality 8. Some of the variation may be explained by the varying study designs, study population sizes, and differences in the adjustment for confounding variables. Using data on 5354 BC patients from the King's Health Partners Breast Cancer Biobank (KHPBCBB), we assessed the extent to which family history of BC was associated with BC severity and BC‐specific mortality.

## Methods

Study population and data collection

The King's Health Partners Breast Cancer Biobank consists of over 12,000 patients, added consecutively with prospectively recorded data, who were treated for invasive BC at Guy's Hospital, London between January 1st 1975 and December 31st 2012. For the purposes of this study, we selected all women diagnosed with a primary invasive BC at Guy's Hospital, with data available on their family history. Patients were excluded if there was no documentation of curative surgery being undertaken, and if consent was not obtained. We also excluded 18 women who were lactating or pregnant at time of diagnosis, 1303 women with missing data for family history and 205 with an unknown menstrual status were not included. The final cohort contained 5354 women.

The following demographic and clinical characteristics, recorded prospectively, were included: age (≤40, 40–49, 50–59, 60–69 and ≥70), self‐reported ethnicity (white, black, mixed, and other), socioeconomic status (SES) (scored as low, medium, high), parity (split into categories of 0, 1–2, 3–4, and ≥5 live births), natural menopausal status (pre, peri, and post), clinical tumor size (≤2 cm, 2–5 cm, and ≥5 cm), pathological lymph node status (none, <3, 4–10, and ≥10), histopathological grade, estrogen receptor, progesterone receptor, and HER2 status, and systemic treatments (including chemotherapy, endocrine therapy or radiotherapy). Socioeconomic status (SES) was based on the patients’ postcode using the ‘The English Indices of Deprivation 2010’, which is based on education, income, and wealth of environment**.** Family history was patient reported, and divided into first degree, second degree, first and second degree and finally, third degree and no history of BC. Patients with only a third degree of family history were grouped with those without any family history because evidence is limited, but the existing consensus is that the associated increased risk of BC is smaller in patients with only a third degree relative with a history of BC 1. BC severity was defined using a modified version of the St Gallen criteria, as shown in Table 1 21.

**Table 1:** Breast cancer (BC) severity criteria, created using a modified version of the St Gallen criteria 21

Good	Moderate	Severe
ER (estrogen receptor) + or ER‐*AND*TNM Stage I	ER+, age >40*AND*TNM Stage II	<40 years, TNM Stage II*OR*ER‐, TNM Stage II*OR*TNM Stage III or IV

Clinical follow‐up information was collected for all women as the hospital's standard procedure up until the 31st December 2007. After 2007, low‐risk women were not followed up as per hospital protocol, but only those who later returned to clinic with evidence of further disease were followed up. Women who did not return to the clinic were assumed to have not experienced any disease progression and/or recurrence. All follow‐up information was taken from medical records, which also provided information on cause of death, date of last contact, and date of disease recurrence. National death certificate information was obtained from the National Cancer Registry, and the Office of National Statistics, which were used to confirm the cause of death. Data collection for the purposes of this study was permitted under the approval of the Guy's NHS Research Ethics Committee (REC Number: 12/EE/0493) for women diagnosed up to September 2006, for women diagnosed afterwards individual consent was obtained.

### Statistical analysis

The association between family history and BC severity at the time of diagnosis was analyzed with multivariate proportional odds models, as the latter is an ordinal categorical outcome measurement. All models were checked to ensure that the proportional odds assumption held. As a next step, multivariate Cox proportional hazards models were used to assess the association between family history and BC‐specific death. Follow‐up time was defined from the time of diagnosis until death or last day seen. All models were adjusted for age at diagnosis, time period of diagnosis, ethnicity, SES, parity, menopausal status, positive nodes, tumor size, ER, PR, HER2, as well as primary treatment unless stated otherwise. Stratified analyses were performed by time period of diagnosis (1975–1989 or 1989–2012). This stratification aimed to capture the effects of the introduction of mammography screening 22. All statistical analyses were performed with Statistical Analysis Systems (SAS) release 9.3 (SAS Institute, Cary, NC).

## Results

Of the 5354 included women, 3428 (64%) had information sufficient to categorize them by severity at time of diagnosis. Of these women categorized by severity, 791 (23%) had a family history of BC: patients with only a first degree accounted for 428 (54%), while those with second degree numbered 286 (36%) and finally, first and second degree accounted for 77 (10%). Thus, leaving 1926 women (36%) with either no family history of BC as defined by first or second. Patient characteristics of women with information for severity, and women without are shown in Table 2: there were no significant differences between them. Furthermore, there was no significant difference in the mean age at primary diagnosis between women with a family history (56.04 [SD ± 12]) and women without (654.52 [SD ± 11.9]).

**Table 2:** Descriptive characteristics by family history

	All BC			BC (breast cancer) with Severity Information			No severity information		
	Family History (*N* = 1189) *N* (%)	No Family History (*N* = 4165) *N* (%)	*P*‐value	Family History (*N* = 791) (*N* %)	No Family History (*N* = 2637) (*N* %)	*P* ‐value	Family History (*N* = 398) (*N* %)	No Family History (*N* = 1528) (*N* %)	*P* ‐value
Age at diagnosis (years)									
≤40	128 (10.77)	380 (9.12)	0.03	92 (11.63)	280 (10.62)	0.01	36 (9.05)	100 (6.54)	0.53
40–49	297 (24.98)	958 (23.00)		204 (25.79)	600 (22.75)		93 (23.37)	358 (23.43)	
50–59	337 (28.34)	1142 (27.42)		232 (29.33)	726 (27.53)		105 (26.38)	416 (27.23)	
60–69	264 (22.20)	986 (23.67)		182 (23.01)	249 (24.61)		82 (20.60)	337 (22.05)	
≥70	163 (13.71)	699 (16.78)		81 (10.24)	382 (14.49)		82 (20.60)	317 (20.75)	
Socioeconomic status									
Low	702 (59.04)	2607 (62.59)	0.02	487 (61.57)	1727 (65.49)	0.03	215 (54.02)	880 (57.59)	0.44
Medium	46 (3.87)	115 (2.76)		39 (4.93)	83 (3.15)		7 (1.76)	32 (2.09)	
High	256 (21.53)	781 (18.75)		178 (22.50)	528 (20.02)		78 (19.60)	253 (16.56)	
Missing	185 (15.56)	662 (15.89)		87 (11.00)	299 (11.34)		98 (24.62)	363 (23.76)	
Ethnicity									
White	331 (27.84)	924 (22.18)	0.0003	251 (31.73)	721 (27.34)	0.03	16 (4.02)	203 (13.29)	0.007
Black	15 (1.26)	89 (2.14)		8 (1.01)	55 (2.09)		7 (1.76)	34 (2.23)	
Other	43 (3.62)	164 (3.94)		27 (3.41)	106 (4.02)		80 (20.10)	58 (3.80)	
Missing	800 (67.28)	2988 (71.74)		505 (63.84)	1755 (66.55)		295 (74.12)	1233 (80.69)	
Menopausal status									
Pre	433 (36.42)	1459 (35.03)	0.57	305 (38.56)	959 (36.37)	0.34	128 (32.16)	500 (32.72)	0.95
Peri	94 (7.91)	315 (7.56)		61 (7.71)	184 (6.98)		33 (8.29)	131 (8.57)	
Post	662 (55.68)	2391 (57.41)		425 (53.73)	1494 (56.66)		237 (59.55)	897 (58.70)	
Parity									
0	239 (22.27)	853 (22.88)	0.41	170 (23.48)	508 (21.45)	0.24	69 (19.77)	345 (25.37)	0.13
1–2	538 (50.14)	1878 (50.38)		364 (50.28)	1219 (51.48)		174 (49.86)	659 (48.46)	
3–4	258 (24.04)	831 (22.29)		168 (23.20)	534 (22.55)		90 (25.79)	297 (21.84)	
≥5	38 (3.54)	166 (4.45)		22 (3.04)	107 (4.52)		16 (4.58)	59 (4.34)	
Tumor size (cm)									
Unknown	11 (0.96)	32 (0.80)	0.12	0 (0.00)	1 (0.04)	0.45	11 (3.13)	31 (2.27)	0.04
≤2	647 (56.61)	2129 (53.20)		503 (63.59)	1671 (63.37)		144 (40.91)	458 (33.55)	
2–5	444 (38.85)	1655 (41.35)		259 (32.74)	835 (31.66)		185 (52.56)	820 (60.07)	
≥5	41 (3.59)	186 (4.65)		29 (3.67)	130 (4.93)		12 (3.41)	56 (4.10)	
Number of positive lymph nodes									
0	477 (51.46)	1601 (50.05)	0.61	429 (54.24)	1364 (51.73)	0.53	48 (35.29)	237 (42.17)	0.32
1–3	294 (31.72)	999 (31.23)		257 (32.49)	875 (33.18)		37 (27.21)	124 (22.06)	
4–10	93 (10.03)	365 (11.41)		79 (9.99)	296 (11.22)		14 (10.29)	69 (12.28)	
≥10	63 (6.80)	234 (7.31)		26 (3.29)	102 (3.87)		37 (27.21)	132 (23.49)	
Invasive grade									
Unknown	314 (25.41)	1184 (28.43)	0.24	92 (11.63)	320 (12.14)	0.76	222 (55.78)	864 (56.54)	0.62
Grade 1	127 (10.68)	453 (10.88)		107 (13.53)	378 (14.33)		20 (5.03)	75 (4.91)	
Grade 2	420 (35.32)	1344 (32.27)		345 (43.62)	1095 (41.52)		75 (18.84)	249 (16.30)	
Grade 3	328 (27.59)	1184 (28.43)		247 (31.23)	844 (32.01)		81 (20.35)	340 (22.25)	
Estrogen receptor status									
Negative	245 (20.61)	973 (23.36)	0.13	134 (16.94)	475 (18.01)	0.79	111 (27.89)	498 (32.59)	0.17
Positive	739 (62.15)	2485 (59.66)		629 (79.52)	2069 (78.46)		110 (27.64)	416 (27.23)	
Missing	205 (17.24)	707 (16.97)		28 (3.54)	93 (76.86)		177 (44.47)	614 (40.18)	
Progesterone receptor status									
Negative	411 (34.57)	1545 (37.09)	0.18	285 (36.03)	997 (37.81)	0.57	126 (31.66)	548 (35.86)	0.29
Positive	565 (47.52)	1859 (44.63)		469 (59.29)	1508 (57.19)		96 (24.12)	351 (22.97)	
Missing	213 (17.91)	761 (18.27)		37 (4.68)	132 (5.01)		176 (44.22)	629 (78.14)	
HER2‐status									
Negative	486 (40.87)	1642 (39.42)	0.64	375 (47.41)	1152 (43.69)	0.17	41 (10.30)	175 (11.45)	0.16
Positive	127 (10.68)	468 (11.24)		86 (10.87)	293 (11.11)		111 (27.89)	490 (32.07)	
Missing	576 (48.44)	2055 (49.34)		330 (41.72)	1192 (45.20)		246 (61.81)	863 (56.48)	
Surgery type									
Mastectomy	656 (55.17)	2434 (58.44)	0.11	430 (54.36)	1565 (59.35)	0.03	226 (56.78)	869 (56.87)	0.99
Breast conservation	532 (44.74)	1725 (41.42)		361 (45.64)	1070 (40.58)		171 (42.96)	655 (42.87)	
Other surgery	1 (0.08)	6 (0.14)		0 (0.00)	2 (0.08)		1 (0.25)	4 (0.26)	
Chemotherapy									
Yes	270 (22.71)	828 (19.88)	0.03	194 (24.53)	598 (22.68)	0.28	76 (19.10)	230 (15.05)	0.05
No	919 (77.29)	3337 (80.12)		597 (75.47)	2039 (77.32)		322 (80.90)	1298 (84.95)	
Radiotherapy									
Yes	595 (50.04)	1923 (46.17)	0.02	398 (50.32)	1216 (46.11)	0.04	197 (49.50)	707 (46.27)	0.25
No	594 (49.96)	2242 (53.83)		393 (49.68)	1421 (53.89)		201 (50.50)	821 (53.73)	
Endocrine therapy									
Yes	421 (35.41)	1409 (33.83)	0.31	321 (40.58)	1024 (38.83)	0.38	100 (25.13)	385 (25.20)	0.98
No	768 (64.59)	2756 (66.17)		470 (59.42)	1613 (61.17)		298 (74.87)	1143 (74.80)	
BC‐Death									
Yes	345 (29.02)	1335 (32.05)	0.05	215 (27.18)	760 (28.82)	0.37	130 (32.66)	575 (37.63)	0.07
No	844 (70.98)	2830 (67.95)		576 (72.82)	1877 (71.18)		268 (67.34)	953 (62.37)	

Table 3 shows the study population's demographics stratified by BC severity status. No significant differences were observed between women with and without a family history. As a next step, the association between family history and BC severity (at time of diagnosis) was assessed through proportional odds ratios (OR) (Table 4). No ORs observed were statistically significant. For example, when comparing women with any family history to women without family history the proportional OR was 1.00 (95% CI: 0.85–1.17). To investigate this association further, analyses were stratified by time period of diagnosis, but this did not alter any of the findings (Table 4).

**Table 3:** Descriptive table of breast cancer prognosis (at the time of diagnosis, as defined in the methods) by family history of breast cancer as well as diagnosis period

	Good prognosis *N* (%)	Moderate prognosis *N* (%)	Poor prognosis *N* (%)	*P* ‐value
All women with severity (*N* = 3428)	*N* = 2174	*N* = 954	*N* = 300	
Family history				
No	1671 (76.86) (18.76%)	734 (76.94)	232 (77.33)	0.98
Yes	503 (23.14)	220 (23.06)	68 (22.67)	
Degree				
No	1671 (76.86) (18.76%)	734 (76.94)	232 (77.33)	0.42
st degree	270 (12.42)	129 (13.52)	29 (9.67)	
First & Second degree	48 (2.21)	19 (1.99)	10 (3.33)	
Second degree	185 (8.51)	72 (7.55)	29 (9.67)	
Women Diagnosed ≤1989 (*N* = 1,766)	*N* = 1129	*N* = 490	*N* = 147	
Family history				
No	889 (78.74)	377 (76.94)	116 (78.91)	0.71
Yes	240 (21.26)	113 (23.06)	31 (21.09)	
Degree				
No	889 (78.74)	377 (76.94)	116 (78.91)	0.59
First degree	141 (12.49)	64 (13.06)	13 (8.84)	
First & Second degree	15 (1.33)	7 (1.43)	4 (2.72)	
Second degree	84 (7.44)	42 (8.57)	14 (9.52)	
Women diagnosed >1989 (*N* = 1,662)	*N* = 153	*N* = 1045	*N* = 464	
Family history				
No	782 (74.83)	357 (76.94)	116 (75.82)	0.68
Yes	263 (25.17)	107 (23.06)	37 (24.18)	
Degree				
No	782 (74.83)	357 (76.94)	116 (75.82)	0.40
First degree	129 (12.34)	65 (14.01)	16 (10.46)	
First & Second degree	33 (3.16)	12 (2.59)	6 (3.92)	
Second degree	101 (9.67)	30 (6.47)	15 (9.80)	

**Table 4:** Proportional odds ratios (OR) and 95% confidence intervals (CI) by breast cancer severity at diagnosis, for women in the GSTT database, adjusted for time period of diagnosis, ethnicity, and socioeconomic status

	Good, moderate or severe BC (breast cancer)	
	*N* (%)	Proportional OR (95% CI)
All women (*N* = 3428)		
Family history		
No	2637 (76.93)	1.00 (ref)
Yes	791 (23.07)	0.98 (0.84–1.16)
Degree		
No	2637 (76.93)	1.00 (ref)
First degree	428 (12.49)	0.97 (0.76–1.25)
First & Second degree	77 (2.25)	0.98 (0.79–1.20)
Second degree	286 (8.34)	1.07 (0.68–1.70)
Women diagnosed ≤1989 (*N* = 1766)		
Family history		
No	1382 (78.26)	1.00 (ref)
Yes	384 (21.74)	1.06 (0.84–1.33)
Degree		
No	1382 (78.26)	1.00 (ref)
First degree	218 (12.34)	1.21 (0.85–1.72)
First & Second degree	26 (1.47)	0.93 (0.69–1.26)
Second degree	140 (7.93)	1.40 (0.65–2.99)
Women diagnosed >1989 (*N* = 1662)		
Family history		
No	1255 (75.51)	1.00 (ref)
Yes	407 (24.49)	0.91 (0.72–1.15)
Degree		
No	1255 (75.51)	1.00 (ref)
First degree	210 (12.64)	0.99 (0.73–1.33)
First & Second degree	51 (3.07)	0.79 (0.55–1. 13)
Second degree	146 (8.78)	0.96 (0.54–1.70)

Finally, the hazard ratio (HR) for the risk of fatal BC was investigated (Table 5). No association between FH‐ and BC‐specific death was observed (e.g., crude model HR: 0.95 [95% CI: 0.90–1.01]). Similarly, the same was observed when varying degrees of family history were investigated individually (e.g., HR for women with first degree of family history: 0.99 (95% CI: 0.93–1.05) adjusted for age, ethnicity, SES, parity, menopausal status, invasive grade, positive nodes, tumor size, PR, ER, HER2 receptors, surgery type, and adjuvant treatments).

**Table 5:** Hazard ratio (HR) and 95% confidence intervals (CI) for fatal BC (breast cancer) by family history of breast cancer, in women with sufficient data to allow classification by severity

	Fatal	Non‐Fatal	HR (95% CI)^a^	HR (95% CI)^b^	HR (95% CI)^c^
	*N* (%)				
All women (*N* = 6596)	*N* = 975	*N* = 2453			
Family history					
No	760 (77.95)	1877 (76.52)	1.00 (ref)	1.00 (ref)	1.00 (ref)
Yes	215 (22.05)	576 (23.48)	0.95 (0.90–1.01)	0.97 (0.91–1.03)	0.99 (0.93–1.05)
Degree					
No	760 (77.95)	1877 (76.52)	1.00 (ref)	1.00 (ref)	1.00 (ref)
First degree	108 (11.08)	320 (13.05)	0.88 (0.76–1.03)	0.91 (0.78–1.06)	0.96 (0.82–1.12)
First & Second degree	21 (2.15)	56 (2.28)	0.91 (0.64–1.29)	0.94 (0.66–1.34)	0.94 (0. 6–1.34)
Second degree	86 (8.82)	200 (8.15)	0.94 (0.78–1.13)	0.99 (0.82–1.19)	1.02 (0.85–1.23)

a Crude model.

b Adjusted for age, time period of diagnosis, ethnicity, and socioeconomic status (SES).

c Adjusted for age, time period of diagnosis, ethnicity, SES, parity, menopausal status, invasive grade, positive nodes, tumor size, PR, ER (estrogen receptor), HER2 receptors, surgery type and adjuvant treatments.

## Discussion

Given contradicting findings within the existing literature regarding associations between family history and both tumor characteristics and BC‐specific death, we used our hospital‐based cohort to investigate this further. The results from this study demonstrated that family history does not appear to have an association with BC severity at time of diagnosis, nor BC‐mortality.

Literature indicates women with an early‐onset BC with family history have a higher frequency of *BRCA1* and *BRCA2* compared to women without a family history 23, 24. Accumulating evidence has shown that different tumor characteristics were identified in patients with the *BRCA1* mutation – less so in patients with the *BRCA2* mutation ‐ in comparison to patients without the mutation 25, 26, 27, 28, 29, 30. Survival advantage for BC cases with a *BRCA1* mutation has been reported 31, 32, whereas another study found poor survival when the patient's had *BRCA1* mutation 33.

There is discrepancy in the associations between family history and BC prognosis reported to date. A study incorporating Utah's Population Database found that women under the age of 50 with a family history of BC were statistically significantly at a greater risk of a poorer survival when compared to those women without; with a relative risk of 1.54 (95% CI: 0.98–2.41) for women with first degree relatives previously diagnosed with BC 34. In contrast, a recent population‐based study 35 based in Netherlands reported a lower risk of all‐cause death with ≥2 first‐degree relative with family history (HR 0.2; 95% CI 0.06–1.0). Nevertheless, several studies have found no association between family history and BC overall survival 8, 36, 37. For instance, a study based on a Canadian population‐based familial breast cancer registry 38 reported that a family history of breast or ovarian cancer was not associated with recurrence or death. Chang and colleagues also reported a null association with all‐cause death in a cohort of BC patients in the Breast Cancer Family Registry in Northern California, USA when excluding those with BRCA1 and BRCA2 mutation 8. Therefore, findings from our study are also in agreement with the majority of existing research regarding BC‐mortality. Of the studies demonstrating a statistical difference in mortality, most of them focused on younger women, and tended to have smaller study populations.

With regard to disease‐free survival, recent findings from the UK Prospective Outcomes in Sporadic versus Hereditary breast cancer (POSH) suggested that there is no difference in disease progression between female BC patients with or without a family history, with a HR of 0.89 (95% CI: 0.76–1.03) 39. It is clinically relevant to know that women with a family history of BC are not necessarily at a greater risk. Thus, no evidence is provided to date to suggest that these women should be given treatment different to that of what current protocol dictates.

The strength of this hospital‐based cohort is the ability to adjust for possible confounding. For example, adjustments could be made to account for more aggressive treatments, or any confounding by socioeconomic status or ethnicity. Furthermore, while the cohort is hospital based, it is defined by a specific catchment area so that the included individuals remain unselected and it is close to a population‐based cohort. A study based largely on referred individuals, may have led to a bias of different family history. In previous studies where positive associations were observed between FH and severity or mortality limited adjustments for confounding were made 1, 4, 5, 14, 18. By contrast, studies which observed no association between the two, as seen here, adjusted for established prognostic factors along with age, and race in some instances 8, 9, 10, 37.

Despite its large size, there are some limitations to consider. Firstly, patient genetics were not collected in our study. Thus, we cannot disentangle any effect modifications by type of mutation (i.e., *BRCA1*). Secondly, approximately one‐third of women in the dataset did not have information on BC severity recorded. However, it can be seen in Table 2 that the women with severity are representative of the entire cohort. A further limitation of this dataset is the incomplete data collection for some confounding variables, such as ER/PR/HER2 status. However, due to the large population and extensive follow‐up period, it remains a valuable and informative resource. Another potential limitation of this dataset is the lack of additional information for the known risk factors of BC, such as age at menarche, age at first birth, obesity, and HRT. However, these risk factors (bar obesity) are not among those considered to be strong prognostic factors of BC and thus we deem the potential confounding influence to be low 40, 41, 42. In terms of disease severity, a modified version of the St Gallen's criteria was used where we incorporated information on age (above or below 40 years at the time of diagnosis), ER status as well as histopathological grade. A strength here is that these elements were prospectively derived from our KHPBCBB database. Furthermore, while only invasive tumors were included, we did not stratify by individual tumor type due to power issues resulting from smaller numbers. Finally, the long‐time span of recruitments could be viewed as a potential variable to introduce bias, however, we believe that by adjusting for potential confounders such as treatment, surgery, age and diagnosis, and the diagnosis period, that the potential for any influence on the findings is small.

### Conclusion

We did not find evidence to support an association between family history of BC and severity and BC‐specific mortality. Our results indicate that clinical management should not differ between women with and without family history when the underlying mutation(s) is (are) unknown. The literature indicates that it may be different for *BRCA1/2* carriers.

## Conflict of Interest

None declared.
